# The Cycling of Intracellular Calcium Released in Response to Fluid Shear Stress Is Critical for Migration-Associated Actin Reorganization in Eosinophils

**DOI:** 10.3390/cells10010157

**Published:** 2021-01-15

**Authors:** Kiho Son, Amer Hussain, Roma Sehmi, Luke Janssen

**Affiliations:** Department of Medicine, McMaster University, Hamilton, ON L8S 4L8, Canada; hussaa1@mcmaster.ca (A.H.); sehmir@mcmaster.ca (R.S.); janssenl@mcmaster.ca (L.J.)

**Keywords:** actin, calcium signaling, confocal microscopy, integrin, mechanosensing, membrane ruffles, pseudopodia, shear stress

## Abstract

The magnitude of eosinophil mobilization into respiratory tissues drives the severity of inflammation in several airway diseases. In classical models of leukocyte extravasation, surface integrins undergo conformational switches to high-affinity states via chemokine binding activation. Recently, we learned that eosinophil integrins possess mechanosensitive properties that detect fluid shear stress, which alone was sufficient to induce activation. This mechanical stimulus triggered intracellular calcium release and hallmark migration-associated cytoskeletal reorganization including flattening for increased cell–substratum contact area and pseudopodia formation. The present study utilized confocal fluorescence microscopy to investigate the effects of pharmacological inhibitors to calcium signaling and actin polymerization pathways on shear stress-induced migration in vitro. Morphological changes (cell elongation, membrane protrusions) succeeded the calcium flux in untreated eosinophils within 2 min, suggesting that calcium signaling was upstream of actin cytoskeleton rearrangement. The inhibition of ryanodine receptors and endomembrane Ca^2+^-ATPases corroborated this idea, indicated by a significant increase in time between the calcium spike and actin polymerization. The impact of the temporal link is evident as the capacity of treated eosinophils to move across fibronectin-coated surfaces was significantly hampered relative to untreated eosinophils. Furthermore, we determined that the nature of cellular motility in response to fluid shear stress was nondirectional.

## 1. Introduction

Eosinophils are a subset of granulocytes that play a vital role in the inflammatory processes associated with a number of airway diseases including asthma [1], eosinophilic granulomatosis with polyangiitis (EGPA) [2], and hypereosinophilic syndrome (HES) [3]. As the positive correlation between eosinophil accumulation in pulmonary tissues and disease severity has been ascertained [4], the past decade has seen the development of several biologics that target interleukin-5 (IL-5), a T_H_2 cytokine largely responsible for the mobilization, recruitment, and survival of eosinophils at sites of inflammation [5]. Although some of these anti-IL-5 clinical trials have reported effective reduction and/or depletion of eosinophils from the blood and airways of severe asthmatic patients [5,6], it has become a point of interest to investigate other factors such as epithelial cell-derived cytokines (IL-25, TSLP) and IL-13-secreting type 2 innate lymphoid cells (ILC_2_) that may also contribute significantly to eosinophil migration [5,7,8]. It is vital to untangle the series of signaling events that lead to specific actin cytoskeleton reorganization, which allow for (1) cellular adhesion to the endothelium and (2) transmigration through the extracellular matrix into the surrounding tissues by utilizing membrane protrusions such as pseudopodia.

In the current model of eosinophil extravasation, inactive integrins expressed on the surface of circulating eosinophils will undergo a conformational change to a highly active state upon exposure to chemokines (e.g., eotaxin) near the bronchial vascular endothelium [9,10]. In particular, the very late antigen-4 (VLA-4, α4β1) integrin on the eosinophilic cell surface will bind to the vascular cell adhesion molecule-1 (VCAM-1) counter ligand, which is upregulated in the asthmatic lung [10,11,12]. Furthermore, in vivo pre-activation and/or priming of the cells mediated by increased IL-5 levels in asthmatics result in intermediate-activated integrin conformations displayed on the surface [9], resulting in augmented integrin activation; extravasation from circulation; and an overaccumulation of eosinophil populations in respiratory tissues, oftentimes leading to subsequent airway inflammation and obstruction.

Our previous study identified a novel phenomenon in eosinophils, which we coined the perfusion-induced calcium response (PICR), in which the perfusion of media without any pharmacological agents over adhered eosinophils from peripheral blood elicited an integrin-mediated release of intracellular calcium [Ca^2+^]_i_ from internal stores. The calcium flux was observed concurrently with changes in eosinophil morphology (flattening, membrane protrusions) [13], suggesting the following: (1) the signaling pathways involved in actin skeleton restructuring may be regulated by [Ca^2+^]_i_ mobilization, and (2) mechanical stimuli of fluid shear stress detected by integrin mechanosensors on the eosinophil surface may play a pivotal role in eosinophil migration from the vasculature. Indeed, eosinophils in vivo would experience varying magnitudes of shear stress corresponding to their strength of adhesion to the vascular endothelium, in turn triggering the PICR under optimal environmental conditions. In a subsequent study, we observed, in response to physiologically-relevant fluid shear stress levels, a temporal link between [Ca^2+^]_i_ release and increased cell surface contact area with the fibronectin-coated substratum via cell flattening [14]. Unfortunately, at the time, we were neither able to clearly elucidate the correlated/causative nature nor the directionality of this relationship in eosinophils. The present study utilizes improved real-time confocal microscopy techniques and refined analytic methods to investigate the effects of pharmacological inhibitors to calcium and actin cytoskeleton signaling pathways on the PICR in eosinophils. Improving upon the limited knowledge regarding eosinophil trafficking processes may assist in the future development of novel treatments and therapies of eosinophilia-driven pathologies such as asthma.

## 2. Materials and Methods

### 2.1. Ethics Statement

Healthy adult volunteers (*n* = 8) were recruited with written consent to participate in this study. The institutional ethics committee (Hamilton Integrated Research Ethics Board; HIREB) granted ethical approval to obtain blood samples from participants (McMaster University REB Project #12-3764). The inclusion criteria for donors were as follows: no history of asthma, not on any type of medication, and no illness prior to blood donation.

### 2.2. Eosinophil Isolation

The MACSxpress Eosinophil Isolation Kit (catalogue #: 130-104-446; Miltenyi Biotec Inc., Bergisch Gladbach, Germany) was utilized to isolate eosinophils by negative selection from whole blood draws as previously outlined in detail per Son et al. [15]. Briefly, the kit contains a cocktail of magnetically labelled antibodies conjugated to various surface markers of non-eosinophilic cells. This solution is mixed with 30 mL of peripheral blood samples collected into EDTA anticoagulant vacutainers (BD Bioscience, San Jose, CA, USA), and incubated for 15 min within a specialized magnet to allow for segregation. Residual erythrocytes in the enriched population were lysed with 9 mL of sterile endotoxin-free water for 20 s (hypotonic lysis) and topped off with 1 mL of 10× HBSS media without calcium and magnesium (Invitrogen, Carlsbad, CA, USA). The isolated eosinophil populations (purity: 95.0 ± 1.7%; viability: 95.3 ± 4.4%) [15] were resuspended in RPMI1640 (0.42 mM calcium) at 10^6^ cells/mL and kept at 4 °C for a minimum of 2 h to allow pre-activated or primed eosinophils to return to an unstimulated, baseline state. Experiments were run within 12 h of isolation to avoid defective cell function as a result of viability loss over time.

### 2.3. Confocal Fluorescence Microscopy

Glass coverslips (40 mm diameter, 1.5 mm thick; Warner Instruments, Hamden, CT, USA) were cleaned in 55 °C 1M HCl overnight, and then washed twice in distilled water and twice more in double distilled water for 10 min each. Afterwards, the coverslips were rinsed with 100% ethanol and allowed to dry before storing them in a clean container for future use. The fibronectin solution (Sigma Aldrich, St. Louis, ON, Canada) was prepared at 2 µg/mL in PBS, of which 1 mL was pipetted onto an aforementioned acid-washed coverslip resulting in an approximately 4 µg/cm^2^ coating when dried overnight.

A low-profile parallel plate flow chamber (RC-31; Harvard Apparatus, Holliston, MA, USA) was utilized as the experimental apparatus for confocal imaging. The bath chamber could be disassembled into top and bottom halves and screwed back together while sandwiching a silicon gasket with a vertical height of 125 µm to represent the side walls. Eosinophils transferred onto the fibronectin-coated coverslip would be placed securely on top of the bottom half of the chamber prior to assembly. The perfusate was introduced through a peristaltic pump (PS-200; Living Systems Instruments, St Albans, VT, USA) at a perfusion rate inducing fluid shear stress at 37.5 dynes/cm^2^, which was within the physiologically relevant range (20–40 dynes/cm^2^) of shear stress experienced by eosinophils migrating from post-capillary venules [16].

A total of 300 µL of the resuspended eosinophil population was loaded with 3 µL of the calcium binding dye, Fluo-3 AM (3.5 µM in DMSO w/0.01% pluronic acid), and incubated in an opaque 1.5 mL tube for 20 min prior to transferring onto the fibronectin-coated coverslips for an additional 40 min incubation in the dark. Where applicable, the eosinophils were incubated for 10 min with various pharmacological agents before loading with Fluo-3 AM. To interfere with the calcium signaling pathways, we treated eosinophils with either ryanodine (10 µM) or cyclopiazonic acid (CPA) (10 µM). The inhibition of actin cytoskeletal reorganization was analyzed with compounds CK-666 (10 µM) and Y-27632 (10 µM). All inhibitor concentrations were previously optimized, and the experiments were conducted at room temperature. The experiment recordings generally took 10 min to complete and were digitized as outlined per Ahmadzai et al. [13].

### 2.4. Signal Processing and Statistical Analysis

The untreated and treated eosinophil recordings were processed as outlined per Son et al. [14]. Briefly, the QuimP plug-in (University of Warwick, Warwick, UK) for the open platform software ImageJ (National Institute of Health, Bethesda, MD, USA) was utilized for .tiff file analysis. The QuimP module allowed images to be transformed into binary black-or-white images by thresholding greyscale pixels to differentiate regions of interest (ROIs) from baseline background noise. The ROIs could be tracked over the course of the experiment to quantify parameters of interest for both morphology (area ratio and adhesion) and fluorescence (intensity and latency of calcium release).

A couple noteworthy improvements have been implemented for the current study. Firstly, a higher magnification was employed for the field of view (FOV), effectively observing fewer cells, but obtaining more precise and accurate measurements of our parameters of interest. As a direct consequence, we were able to expand our outcome variables to include cell elongation, displacement, membrane ruffling, and pseudopodia formation. Furthermore, the higher magnification allowed for the detection for multiple calcium spikes in some eosinophils, a markedly absent feature in our earlier studies. Secondly, we increased the number of images averaged per frame from 10 to 15 to reduce background noise and reduced the frame rate from 2 to 1.5 s per frame to better resolve fleeting changes in cell morphology and fluorescence.

In our previous study [14], area and fluorescence ratios were obtained by averaging values from multiple cells isolated from a given subject on a particular day. However, we noted significant day-to-day variance in certain outcome variables for our untreated control group—not just between different blood donors, but also in eosinophils from a given donor on different days. Therefore, to minimize this variance, outcome measurements for all treatment groups were normalized to the average value of the untreated control group of the corresponding donor and day. For example, in quantifying cell flattening in response to shear stress (calculated by dividing the average area of individual cells over the final 60 s of the recording by the average area of the cell at baseline), the control cells on a given day might exhibit a 53% increase in area, while cells of a given treatment group might exhibit a 38% increase in area; therefore, we recorded a normalized area change in that treatment group on that day of 38/53. This was done for all replicates of treatment groups studied on various days and we report here the mean normalized area change (+/− SD). The same was done in quantifying the fluorometric response (calculated by dividing the peak of the transient spike-elevation by the average of the baseline fluorescence before onset of perfusion). However, we were unable to run all four of our different treatment conditions every single experiment day, because of the low eosinophil population count isolated from healthy donors (~1.5 million cells per 25–30 mL venous blood). As a result, we ran the untreated, control eosinophil group more often (*n* = 6) than the treatment groups (*n* = 4 for each), with approximately 2–5 cells analyzed per experiment.

Graphpad Prism 6 (La Jolla, CA, USA) was used to conduct all statistical analyses and generate figures. Statistical analyses involved Kruskal–Wallis tests to compare means of two or more groups, followed by Dunn’s multiple comparisons post hoc test. Fischer’s exact test was used to compare event frequencies (e.g., membrane ruffling, pseudopodia) of treated cells against the untreated control group. In all cases, *p* < 0.05 was considered to be significant.

## 3. Results

### 3.1. Pharmacological Agents

A total of four pharmacological inhibitors were utilized to disrupt the calcium signaling and actin regulatory pathways. Cyclopiazonic acid (CPA) is a specific inhibitor of sarco(endo)plasmic reticulum Ca^2+^-ATPases, which are responsible for transporting cytoplasmic calcium ions back into the endoplasmic reticulum. Ryanodine binds to ryanodine receptors (RyRs), a family of intracellular calcium release channel proteins, keeping them in closed conformations at micromolar concentrations. With respect to actin cytoskeleton rearrangement, the small molecule CK-666 functions as an allosteric antagonist to the Arp2/3 protein complex, which is a well-known player in actin nucleation. Lastly, the compound Y-27632 binds to the catalytic site of ROCK kinases, which are critical for the actin stabilization and the formation of stress fibres.

### 3.2. Normalizing Treatment Groups to the Control for Area Ratio

**Figure 1:**
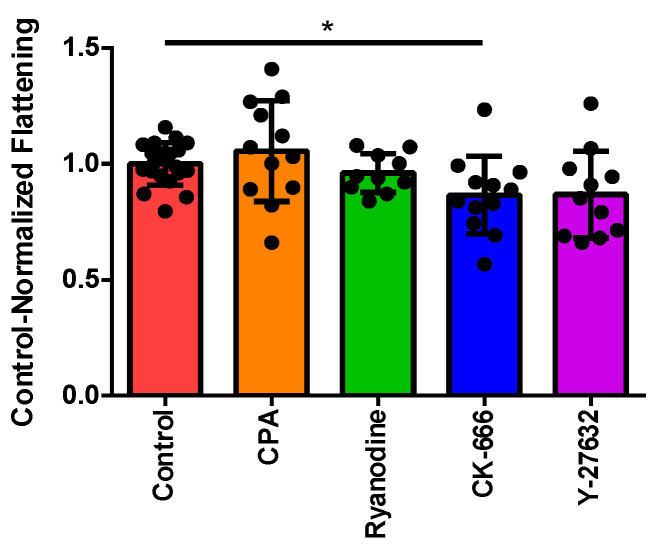
Characterizing changes in cell–substrate contact area in response to pharmacological inhibitors. Each data point represents an individual eosinophil. Control-normalized cell flattening (see Section 2) in cells treated with vehicle (control; *n* = 20), CPA (*n* = 12), ryanodine (*n* = 10), CK-666 (*n* = 12), or Y-27632 (*n* = 11). * *p* < 0.05 by Kruskal–Wallis test.

As previously mentioned, the area ratio was calculated by dividing the average area for each individual cell over the final 60 s of the recorded experiment by the average cell area prior to perfusion. Using the refinements to our imaging and data analysis described in Section 2 (above), we first confirmed the inhibitory effect of CK-666 upon cytoskeletal reorganization that we noted previously, contrasted against the lack of effect of disrupting Ca^2+^-handling using CPA or ryanodine (Figure 1). The Rho-kinase inhibitor Y-27632 also showed a trend toward decreasing the cell flattening, although this did not reach statistical significance.

### 3.3. Characterizing the Effect of Pharmacological Agents on Shear Stress-Induced Calcium Release

**Figure 2:**
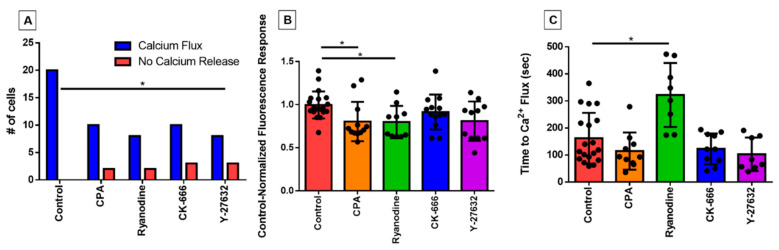
Characterizing changes in perfusion-induced calcium response (PICR) in response to pharmacological inhibitors. (**A**) Numbers of cells exhibiting (blue bars) or not exhibiting (red bars) a PICR under control conditions or in the presence of cyclopiazonic acid (CPA), ryanodine, CK-666, or Y-27632, as indicated. * *p* = 0.037 (Fischer’s exact test). The control-normalized fluorescence response and latency for onset of the calcium-response under those same experimental conditions are given in (**B**,**C**), respectively. Each data point represents an individual eosinophil. * *p* = 0.022 and *p* = 0.002, respectively (Kruskal–Wallis test).

We first investigated whether the inhibitor treatments impacted the occurrence of an intracellular calcium response to shear stress, which is regularly observed in untreated eosinophils. All eosinophils in the control group exhibited the PICR, as expected, evident by a peak fluorescence emission occurring approximately 2 min post-perfusion, whereas a few eosinophils per treatment group did not; the difference was only statistically significant in the case of Y-27632 (Figure 2A). 

In those cells that did show a fluorescent response, the magnitude still represented a dramatic elevation over baseline; previously, we have shown in control cells that this comprises a two- to threefold increase over baseline over the course of a few seconds, followed by a return to baseline over the course of one to two minutes [13,14]. Nonetheless, there was a small reduction in the magnitude of this still very sharp calcium signal, as reflected in mean values of control-normalized fluorescence responses of less than unity (Figure 2B); the reduction was only statistically significant in the case of the two inhibitors of calcium signaling (CPA and ryanodine). More importantly, however, we observed that the time taken for the eosinophil to display this calcium flash was nearly doubled in the ryanodine-treated cells (*p* < 0.05 compared with control; Figure 2C). The time to calcium flash was measured by determining the number of frames post-perfusion taken to reach peak fluorescence emission, and converting the value into seconds (1 frame = 1.5 s). Latency was not statistically changed in the other treatment groups.

### 3.4. Investigating the Effect of Fluid Shear Stress on Loss of Cell Circularity

**Figure 3:**
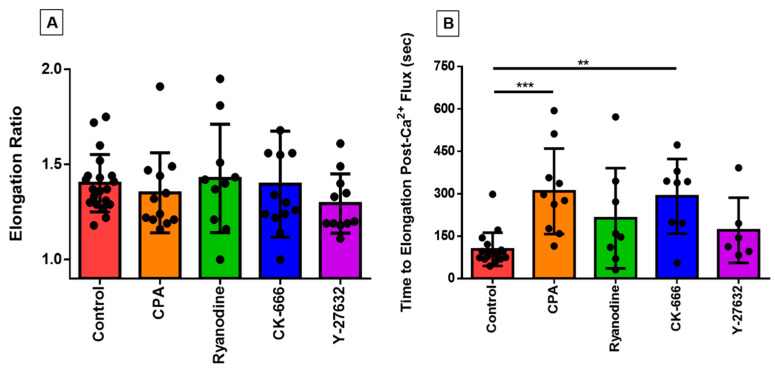
Analyzing the relationship between loss of circularity and the PICR in eosinophils. Each data point represents an individual eosinophil. (**A**) The peak elongation ratio for eosinophils under control conditions or in the presence of CPA, ryanodine, CK-666, or Y-27632, as indicated. (**B**) The time it took for the eosinophil to begin losing circularity after the calcium spike was measured across all treatment groups and compared against the untreated controls (*** *p* < 0.001; ** *p* < 0.01; *p* < 0.0001, Kruskal–Wallis test).

We have previously shown how the eosinophils, which are normally spherical in shape at rest, respond to a variety of excitatory stimuli including eotaxin and shear stress by rapidly flattening and extending cell membrane protrusions in various directions [13,14]; this morphological change takes place over the course of two or three minutes, thus representing the earliest stage of migration and chemotaxis, which have been studied in detail elsewhere [17,18]. We used the QuimP analysis module of ImageJ to quantify this morphological change; this module superimposes a best-fitting ellipse on top of the cellular ROI to track changes in the ellipse’s major and minor axes. In order to ascertain whether the application of pharmacological agents influenced this response, we measured cell elongation by dividing the length of the major axis of the cell by the length of the minor axis, which were both tracked over the duration of the experiment. The highest value in this ratio (which represents peak elongation) was not statistically significant different across treatment groups (Figure 3A).

In contrast, certain of the inhibitors tested here markedly changed the time-course of this morphological response to shear stress, as reflected in an increased latency between peak calcium-response and peak elongation (Figure 3B). This increase was highly statistically significant in the case of CK-666 and CPA, but not for Y-27632 or ryanodine (although there was a trend toward an increase in the latter group).

### 3.5. Pseudopodia Formation and Membrane Ruffling

**Figure 4:**
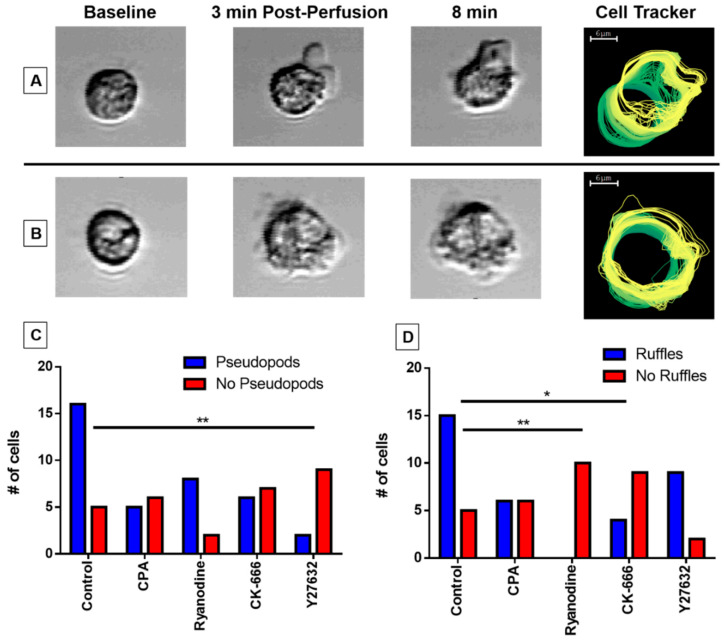
Assessing morphological changes in response to fluid shear stress. Eosinophils may exhibit distinct actin cytoskeletal protrusions in response to fluid shear stress. (**A**) Pseudopodia extension generally precedes cellular movement in the matching direction, whereas (**B**) membrane ruffles maintain membrane circularity and do not associate with cell motility. The cell tracker image (right) traces the cell membrane over the course of the experiment with a green-to-yellow color transition indicator. The capacity of eosinophils to develop pseudopodia (**C**) or membrane ruffling (**D**) was observed under control or treated conditions with CPA, ryanodine, CK-666, or Y-27632. Significance was calculated at (**C**) ** *p* = 0.0017 (Y-27632), (**D**) ** *p* = 0.0001 (ryanodine), and * *p =* 0.012 (CK-666), respectively (Fischer’s exact test).

We observed two distinct morphological features of untreated eosinophils subjected to perfusion-induced shear stress: (1) pseudopodia, in which the membrane morphs non-circularly and extends, often preceding preliminary cell movement in the matching direction (Figure 4A); and (2) membrane ruffling, during which the cell membrane remains circular and intact, with small actin protrusions observed just beyond the membrane (Figure 4B). We noted that the application of our inhibitors impacted the formation of these morphological features. To examine the effect of our agents, we counted whether pseudopodia (Figure 4C) and membrane ruffling (Figure 4D) were formed for individual cells in each treatment group and compared the frequency distribution against the control group. The treatment of Y-27632 (*p* < 0.01) impeded pseudopodia formation (Figure 4C), whereas CK-666 (*p* < 0.05) and ryanodine (*p* < 0.01) impacted the formation of membrane ruffling (Figure 4D).

### 3.6. Directionality of Eosinophil Motility

**Figure 5:**
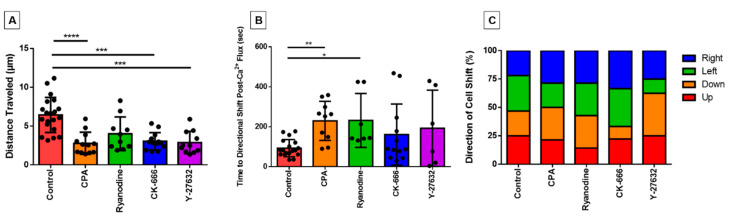
Evaluating eosinophil motility response and its relationship with the PICR. (**A**) The centroid of analyzed eosinophils was tracked over the course of the experiment to determine the total distance traveled (µm) for control and treated (CPA, ryanodine, CK-666, and Y-27632) eosinophils. (*** *p* < 0.001; **** *p* < 0.0001) (**B**) A number of eosinophils changed their direction of movement subsequent to the calcium flash. The time in between the events was measured across treated eosinophils and compared with control eosinophils (* *p <* 0.05; ** *p* < 0.01; *p =* 0.008, Kruskal–Wallis test). (**C**) Percentage of cells in each group that altered their movement path towards one of four different directions (perfusion moves from left to right in the field of view (FOV)).

Although the duration of these experiments is too brief to document bone fide migration, we observed that some cells made initial movements across the surface of the fibronectin-coated coverslip throughout the course of the 10 min post-perfusion. To investigate the effect of our inhibitors on eosinophil motility, we measured the movement of the centroid location for each cell over the course of the experiment to assess the distance traveled. We found that the distances travelled by cells treated with cytoskeleton inhibitors (CK-666; Y-27632) or with the calcium reuptake inhibitor CPA were highly statistically significantly less than control cells (Figure 5A). The ryanodine-treated group also showed lower distance traveled, but this effect was not statistically significant (Figure 5A).

In addition, we assessed the latency between the calcium flash and its succeeding directional shift. We found that, similarly to elongation, that latency was significantly longer in cells treated with calcium inhibitors (Figure 5B). Although the actin signaling inhibitors also had longer time periods between events, the latter were not statistically significantly different (Figure 5B).

Furthermore, we were interested in whether the direction the eosinophil membrane was protruding (and presumably crawling) towards relative to their centroid position was affected by the direction that the perfusion was applied. Although the media was perfused from left to right in our microscopy field of view for the video recordings, the cells did not show a directional preference of actin cytoskeletal protrusion and/or movement (Figure 5C).

## 4. Discussion

The modifications to the cytoskeletal architecture due to stimuli ranging from mechanical fluid shear stress to ligand binding chemokines occur in a systematic fashion to ensure proper spatial and temporal conditions are met for migration-related behavior. Yet, the molecular processes of eosinophil extravasation detailing the activation of its integrin receptors and the subsequent outside-in signaling transduction cascades have not been fully unraveled. Although many groups are investigating the migrational responses triggered and guided by molecular stimuli (chemotactic and chemokinetic ligands), we are pioneering a role for physical stimuli (shear stress) in triggering the extravasation of activated eosinophils from the circulation and into the vessel wall. That is, shear stress elicits a complex and carefully choreographed sequence of morphological changes, which all take place within 10 min, and sets the stage for the migrational response, which occurs over the next many hours. Furthermore, we are finding that the immediate triggering event is connected to intracellular calcium fluxes. In the present study, we disrupt both actin cytoskeleton and calcium pathways with pharmacological agents to probe the eosinophil response to physiologically relevant shear stresses experienced by the cell once it adheres to the vessel wall.

To link the two crucial steps in classically studied leukocyte extravasation of adhesion and membrane protrusion, we utilized fibronectin as our substrate coating. It is well documented that firm cell adhesion to the endothelium and the extracellular matrix is primarily mediated through integrin engagement. The VLA-4 integrin on the eosinophil surface not only binds to VCAM-1 upregulated on the endothelium in disease models [19], but also readily adheres to fibronectin [20], allowing our experimental model to better simulate in vivo endothelium environments. Integrin activation has been shown to activate Rac1, which in turn signals to the Arp2/3 complex [21], a seven-subunit protein aggregate necessary for actin nucleation and actin filament polymerization for numerous essential cell functions including vesicle trafficking, migration, and membrane protrusions (lamellipodia and pseudopodia) [21,22,23]. Rac1 promotes the linkage between the Arp2/3 complex with vinculin, an adaptor protein that physically links the transmembrane integrin receptor to the actin cytoskeleton [24]. This connection may be further stimulated by PIP_2_, which induces a conformational change in vinculin to expose additional binding sites for the Arp2/3 complex [25]. PIP_2_ itself is also involved in the activated G_q_/11 pathway, which results in IP_3_-mediated calcium release. As such, we were motivated to determine the inhibition of the integrin/Rac1/Arp2/3 pathway, a central component to both cellular adhesion and motility, with the compound CK-666, which would interfere with the eosinophil PICR. We also utilized the compound Y-27632 as a cell-permeable Rho-associated protein kinase (ROCK)-selective inhibitor to reduce actin filament stabilization [26]. The GTPase Rho emerged in the late 20th century as a major facilitator of actin skeleton rearrangement necessary for actin stress fiber formation and cell migration through focal adhesions [26]. ROCK was found to be a key downstream Rho effector molecule specifically responsible for stress fiber formation, and itself inhibits actin filament depolymerization [26].

In our study, both CK-666- and Y-27632-treated eosinophils displayed stunted capabilities with regards to environment exploration and cell motility. The inability of the Arp2/3 complex to transiently bind to vinculin and integrin receptor aggregates because of CK-666 inhibition was markedly reflected in its smaller area ratio (Figure 1B) and lack of membrane ruffling (Figure 4D). However, the CK-666-treated eosinophils boasted a robust and rapid calcium spike (Figure 2B,C), suggesting that intracellular calcium release signaling remained upstream or independent from actin rearrangement. It is interesting to note, however, the perfusion of Y-27632 did not significantly affect the eosinophils’ response to fluid shear stress outside of pseudopodia formation (Figure 4C) and distance traveled (Figure 5A). In fact, eosinophils treated with Y-27632 exhibited all of the hallmark features of the fluid shear stress response indicated by the lack of significant differences in area ratio, fluorescence ratio, and high positive correlations between calcium flux, elongation, and directional shift timepoints. These results indicate that pseudopodia formation requires long-term stability of actin polymerization relative to membrane ruffling, and is a necessary checkpoint for substantial eosinophil motility.

However, calcium signaling inhibitor data support our theory that eosinophil PICR is responsible for triggering actin cytoskeleton rearrangement. The compound ryanodine functions as a full antagonist to ryanodine receptors (RyRs) at micromolar concentrations [27]. Although stimulated RyRs are predominantly known to trigger [Ca^2+^]_i_ release from the sarcoplasmic reticulum in skeletal muscle cells to drive contraction [28], it was recently discovered that RyRs are also expressed on the endoplasmic reticulum of other cell types including immune cells [29]. Subsequent to [Ca^2+^]_i_ release in cells, the Ca^2+^-ATPases on the plasma membrane and endomembrane are responsible for restoring calcium homeostasis by pumping Ca^2+^ into the extracellular space and back into intracellular stores, respectively [30]. Therefore, treating cells with the ER Ca^2+^-ATPase inhibitor, CPA, would deplete calcium stores over time.

Although the CPA-treated eosinophils displayed fluorescence ratios of a smaller magnitude, it did not influence the latency of the calcium flux post-perfusion (Figure 2C). The CPA data strongly suggest that cycling intracellular calcium ions between the cytosol and ER storage is integral to migration-related actin rearrangement. In essence, as a consequence of calcium ion reuptake inhibition, the smaller PICR in this treatment group resulted in weak temporal association with both cellular elongation and directional changes in movement. On the other hand, we surmised that the reason ryanodine-treated eosinophils did not abolish the PICR (Figure 2A), despite taking a significantly longer time achieving it (Figure 2C), was due to the involvement of alternative calcium signaling pathways such as the PLC-IP_3_ pathway.

The acquisition of spatial asymmetry in cells is critical for the onset of cellular migration. In essence, polarized cell morphology with distinguished front and back ends allows the cell to continue the formation of actin polymerization at the leading region with filaments released from adhesions at the rear [31,32]. In order for sustained migration to occur, the membrane protrusions must be firmly adhered to the substrate. If the pseudopodia fail to stabilize adhesions, they will retract towards the cell body [33]. On the other hand, although membrane ruffles lack adhesion sites and cannot promote displacement [34], it has been suggested the ruffles play an indirect role in promoting migration. Membrane ruffling is associated with macropinocytosis, a transient endocytic process that internalizes extracellular components in addition to parts of the cell membrane and their surface receptors [34]. Specifically, the ruffles may be responsible for redistributing the β1 integrins towards newly formed adhesion sites at the leading edge and the establishment of the front-rear-axis of the cell. The necessity for both pseudopodia and membrane ruffling formation in cell ruffling is evident in the ryanodine treatment group. Although these eosinophils had the most comparable degree of distance traveled relative to the control group because of their higher ratio of pseudopodia formation, the complete lack of membrane ruffling appears to be limiting their potential.

The calcium-spike is a very rapid, all-or-none, pan-cellular event; these are all good and useful features of a finely tuned trigger mechanism. Our data suggest that the morphological changes (flattening, ruffling, and pseudopod-formation) can still occur after disruption of this trigger, but their latency is greatly increased. This might offer up a new avenue for treatment strategy for eosinophilic inflammation. That is, in the physiological setting, circulating eosinophils become primed by agents (such as IL-5), then roll along the vessel wall until they are stimulated by a chemoattractant signal (such as eotaxin) to extravasate. The markedly increased shear stress produced during the rolling phase triggers the PICR, which in turn commits the cells to adhere strongly and accelerate extravasation. If that PICR could be disrupted, the primed cells might otherwise continue to roll and eventually be swept away back into the systemic circulation, thus reducing inflammation at that site. In conclusion, we theorize that adequate strength of the PICR ‘jump-starts’ the extravasation process in vivo and plays a significant role in the temporal aspect of migration.

The study of mechanosensors on circulating leukocytes is fairly novel, and the majority of shear stress research remains dominantly limited to non-migratory cells such as the endothelium. However, it is crucial that we begin to differentiate between the role of mechanical stimuli for the various cell types. For example, the β1 integrins highly expressed on endothelial cells (ECs) were recently found to sense unidirectional flow and promote cellular alignment for vascular homeostasis, suggesting a role distinct from the β1 integrins on eosinophils [35]. Furthermore, some studies have shown that mechanical stress on ECs triggered the influx of calcium from extracellular sources [36], whereas our study indicates the increases in cytosolic calcium are from internal stores. In conclusion, investigating the distinct role of integrin mechanosensors in eosinophils and their role in mobilization may have future therapeutic implications for asthma and other eosinophilia-driven conditions.

## Data Availability

The data presented in this study are available on request from the corresponding author.
